# Real-Time Analysis of Endogenous Wnt Signalling in 3D Mesenchymal Stromal Cells

**DOI:** 10.1155/2016/7132529

**Published:** 2016-09-07

**Authors:** Fatima Saleh, Alice Carstairs, S. Leah Etheridge, Paul Genever

**Affiliations:** ^1^Department of Biology, University of York, York YO10 5DD, UK; ^2^Faculty of Health Sciences, Beirut Arab University, P.O. Box 115020, Beirut, Lebanon

## Abstract

Wnt signalling has been implicated in the regulation of stem cell self-renewal and differentiation; however, the majority of* in vitro* studies are carried out using monolayer 2D culture techniques. Here, we used mesenchymal stromal cell (MSC) EGFP reporter lines responsive to Wnt pathway activation in a 3D spheroid culture system to mimic better the* in vivo* environment. Endogenous Wnt signalling was then investigated under basal conditions and when MSCs were induced to undergo osteogenic and adipogenic differentiation. Interestingly, endogenous Wnt signalling was only active during 3D differentiation whereas 2D cultures showed no EGFP expression throughout an extended differentiation time-course. Furthermore, exogenous Wnt signalling in 3D adipogenic conditions inhibited differentiation compared to unstimulated controls. In addition, suppressing Wnt signalling by Dkk-1 restored and facilitated adipogenic differentiation in MSC spheroids. Our findings indicate that endogenous Wnt signalling is active and can be tracked in 3D MSC cultures where it may act as a molecular switch in adipogenesis. The identification of the signalling pathways that regulate MSCs in a 3D* in vivo*-like environment will advance our understanding of the molecular mechanisms that control MSC fate.

## 1. Introduction

Mesenchymal stromal cells (MSCs) are a heterogeneous cell population first isolated from the bone marrow, with other sources of related cell types also present in other tissues, for example, in dental pulp and adipose tissue [1–3]. The MSC population contains stem cells able to differentiate into osteoblasts, adipocytes, and chondrocytes both* in vitro* and* in vivo* [4]; hence the term “mesenchymal stem cell” is often used, although referring to nonclonal undefined cell types. Despite this limitation, MSCs are widely viewed to have a range of therapeutic regenerative applications, particularly in a musculoskeletal setting [5]. By increasing our understanding of the role of signalling pathways in orchestrating the fate of MSCs, we will generate a fundamental basis upon which we can build, to manipulate these cells for novel treatment strategies in a wide range of diseases.

**Figure 1:**
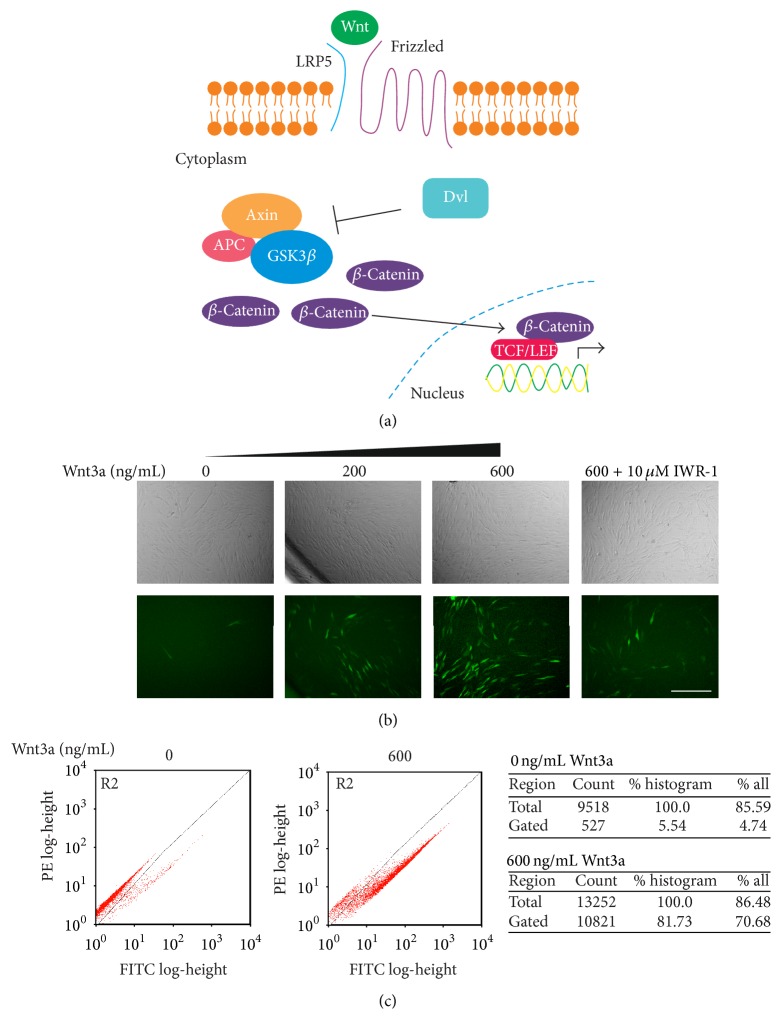
Stimulation using Wnt3a results in observable fluorescence in human Y201 MSC-EGFP reporter lines. (a) Overview of activated canonical Wnt signalling pathway. (b) Fluorescence microscopy of human Y201 MSC-EGFP reporter cells treated with varying concentrations of Wnt3a with or without a Wnt pathway inhibitor, IWR-1. Scale bar = 200 *μ*m. (c) Flow cytometry scatter plot histograms of Wnt3a-treated Y201 Wnt EGFP reporters versus untreated, after removal of dead cells and debris by gating. Shift in population away from autofluorescence indicates positive EGFP expression (lower right quadrant).

One of the regulatory pathways associated with controlling MSC proliferation, differentiation, apoptosis, and migration is the Wnt signalling pathway [6, 7]. Canonical Wnt signalling (Figure 1(a)) is initiated by Wnt ligands binding to membrane receptors Frizzled (Fzd) and coreceptor low-density lipoprotein receptor-related protein (LRP5/6), activating Dishevelled (Dvl). Dvl then inhibits phosphorylation of *β*-catenin by glycogen synthase kinase- (GSK-) 3*β*, leading to *β*-catenin stabilisation and accumulation in the cytosol before its translocation to the nucleus. Nuclear *β*-catenin interacts with members of the lymphoid enhancer factor (LEF) and T-cell factor (TCF) transcription factor family, activating target gene expression [6, 7]. At the receptor level, the Wnt-Fzd receptor interaction may be inhibited by proteins such as Dickkopf (Dkk) [8, 9]. Numerous* in vivo* studies have implicated Wnt signalling as being vital in osteoblast development with both disrupting and activating mutations of LRP5 impacting on bone mass [10–12]. It is also evident that all the components for Wnt signalling activation exist in MSCs and can be stimulated [6]. Yet, discrepancies exist between* in vivo* data using mouse models and differentiation studies performed* in vitro* where, in multiple instances, the stimulation of Wnt signalling has been shown to inhibit differentiation [13–17].

To address these issues, we employed a three-dimensional (3D) culture model with real-time fluorescent reporters to investigate canonical Wnt signalling in MSCs, including murine MSCs and a highly characterised immortalised human clonal MSC line, under control and differentiated conditions. Although conventional culture methods are important experimental techniques, cells grown in 2D are not exposed to the same environmental conditions as cells in 3D tissues, which include locally acting signalling gradients [18]. In fact, a study has previously shown that Wnt signalling, both canonical and noncanonical, was enhanced in MSC spheroids (in this case formed using a biomaterial derived from chitin) leading to altered differentiation capacities based on activation of the Wnt pathway. This was not apparent in 2D MSC monolayer culture [19]. Unlike many other 3D culture methods, we used a 3D spheroid system that does not rely on attachment to any surface, such as a scaffold; rather, the cells form self-aggregating spheroids in a viscous medium [20]. Using this 3D culture system in combination with Wnt reporter cell lines, we examined endogenous and exogenous Wnt signalling under undifferentiated and differentiated conditions, providing evidence that Wnt signalling in MSCs is context-dependent.

## 2. Methods

### 2.1. Cell Culture in 2D and 3D

The Y201 hTERT-immortalised human clonal MSC line, which we have generated and characterised previously [21], and a mouse MSC line, C3H10T1/2, were cultured in Dulbecco's modified Eagle's medium (DMEM) containing 10% foetal bovine serum (FBS) and maintained at 37°C in a 5% CO_2_/95% air atmosphere. The Y201 cells were used as a model human MSC line, whilst the C3H10T1/2 cells were used as a model murine MSC line to enable observations to be made in MSC-type cells from different, commonly used species involving Wnt-based analyses. Wnt3a (Biotechne) was supplemented where indicated at varying concentrations as a positive control for Wnt pathway activation. The Wnt inhibitor IWR-1 was obtained from Sigma Aldrich.

For 3D culture of both cell types, 30,000 MSCs were seeded into U-bottomed nonadherent 96-well plates in media containing 0.25% (w/v) methylcellulose; complete media changes were performed every 2-3 days throughout the time-course.

### 2.2. Establishing a Wnt-Responsive C3H10T1/2 Cell Line Using a TCF Reporter Construct with EGFP

To construct the reporter plasmid, the human cytomegalovirus (CMV) promoter from expression vector pEGFP-N1 (Clontech) was removed by AseI and HindIII cleavage and replaced with a fragment cut from the SuperTopFlash plasmid (M50) (kind gift from Professor R. Moon, Addgene plasmid # 12456) [22] using KpnI and HindIII. Briefly, pEGFP-N1 and M50 plasmids were linearised with the restriction enzymes AseI and KpnI, respectively, and blunted by DNA Polymerase I Large (Klenow) Fragment (Promega). Linearised plasmids were then purified with a QIAquick PCR Purification Kit (Qiagen) according to the manufacturer's instructions before the plasmids were both cut with HindIII to give rise to a pEGFP-N1 (minus CMV) backbone vector and TCF binding sites/TK promoter (insert). Both the backbone vector and insert were run on a 1% agarose gel and then gel-extracted using the QIAquick Gel Extraction Kit (Qiagen) according to manufacturer's instructions. Prior to ligation with T4 DNA ligase (Promega), the pEGFP-N1 backbone was dephosphorylated using Shrimp Alkaline Phosphatase (SAP) (Promega). The ligated plasmid was then transformed into Hi-Coli-5A chemically competent cells (Advantagen) according to the manufacturer's instructions, plated out on LB/Kanamycin plates and incubated overnight at 37°C. DNA was isolated using the GenElute*™* HP Plasmid Purification Kit (Sigma), and the integrity of the SuperTop-EGFP construct was checked by double digestion with XhoI and HpaI.

Confluent C3H10T1/2 cells were transfected using Lipofectamine 2000 according to manufacturer's instructions (Invitrogen), and the plasmid was linearised prior to transfection using Apal. A positive control with pEGFP-N1 and a mock transfection control were also performed. To select for stable transfectants, 2-3 days after transfection, cells were exposed to 1 mg/mL Geneticin (Calbiochem). Geneticin-containing medium was changed twice a week and, after 2 weeks, cells were cultured in C3H10T1/2 medium containing a reduced concentration of Geneticin (500 *μ*g/mL) to maintain stable transfectants. To detect for stable transfection, cells were treated with 5 *μ*M BIO, an activator of Wnt signalling, or DMSO as a vehicle control.

### 2.3. Establishing a Wnt Pathway Responsive Y201 hTERT MSC Cell Line Using a Cignal Lenti TCF/LEF Reporter

Y201 hTERT MSCs were transduced using the Cignal Lenti TCF/LEF kit (Qiagen) according to manufacturer's instructions. Following transduction, cells were cultured under puromycin selection (10 *μ*g/mL) and serially diluted across a 96-well plate (following Corning protocol) to isolate individual clonal Y201-Wnt hTERT MSC lines. These were evaluated for EGFP expression in response to Wnt3a activation by flow cytometry and responsive lines were selected.

Wnt signalling in the Y201-Wnt hTERT MSC lines was analysed with a Zeiss Lightsheet Z.1. To allow visualisation of spheroids and cell tracking, hTERT MSCs were labelled with cell tracker red (Life Technologies) prior to spheroid formation as per manufacturer's instructions. Briefly, required numbers of cells were aliquoted and centrifuged to remove FBS. Cells were labelled with cell tracker and any excess stain was neutralised and removed. Samples were then embedded in a 1% agarose/PBS gel rod using a capillary and tight fitting plunger. After the agarose polymerised the gel was extruded from the capillary into the surrounding chamber media (PBS) for imaging.

### 2.4. Assessment of Adipogenesis and Osteogenesis

Osteogenic and adipogenic differentiation of Y201 hTERT MSCs and C3H10T1/2 cells were initiated 24 hours after spheroid formation using differentiation cocktails as previously described, namely, supplementation with 5 mM *β*-glycerophosphate, 50 *μ*g/mL L-ascorbic acid phosphate, and 10 nM dexamethasone or 0.5 mM isobutyl-methylxanthine, 1 *μ*g/mL insulin, 100 *μ*M indomethacin, and 1 *μ*M dexamethasone, respectively [6, 21, 23]. At specific time points, spheroids were embedded in OCT (Tissue Tek) and frozen in liquid nitrogen before sectioning using a Leica CM3050S cryostat (Leica Microsystems).

Adipogenesis was assessed by lipid droplet accumulation using Oil Red O staining as previously described briefly [6, 21, 23]; cells were fixed in 4% w/v paraformaldehyde solution before incubation in 60% isopropanol. Lipid droplets were stained in 0.3% Oil Red O (Sigma Aldrich) and washed before imaging using a brightfield microscope.

Osteogenic differentiation was analysed by staining deposited calcium using 40 mM Alizarin red solution (pH 4.2) after fixing in 4% w/v paraformaldehyde. Plates were allowed to dry fully before imaging.

### 2.5. Immunocytochemistry

To determine and confirm EGFP localisation, undifferentiated, osteogenic, and adipogenic spheroids were sectioned before immunostaining with an anti-EGFP, rabbit Alexa Fluor® 488 conjugated antibody (Invitrogen) at 1 : 500 dilution and counterstained with DAPI. Samples were mounted in Vectashield (Vector Laboratories) and examined using LSM 710 confocal microscope (Zeiss).

### 2.6. Analysis of Wnt Signalling during Adipogenesis

C3H10T1/2 Wnt reporter spheroids were treated with 50% Wnt3a-conditioned medium (CM) or 50% control L-cell-CM and Wnt antagonist Dkk-1 (100 ng/mL) (R&D Systems) under adipogenic conditions for 7 days before EGFP expression was detected by confocal microscopy and adipogenic potential evaluated (Multiphoton Zeiss LSM 510). Wnt3a-CM and L-cell-CM were processed from L-Wnt3a cells, a Wnt3a-overexpressing murine cell line, and the control nontransfected L-cells, respectively, as described previously [6].

## 3. Results

### 3.1. Reporter MSCs Respond Specifically to Wnt Pathway Activation in a Dose-Dependent Manner

We selected the Y201 hTERT MSCs for generating the Wnt reporter based on their equal propensity for the trilineage differentiation, typical of an MSC [21]. These cells were transduced using the Cignal Lenti TCF/LEF Reporter (EGFP) Kit where EGFP is expressed under the control of a minimal (m) CMV promoter and repeats of the TCF/LEF transcriptional response element (TRE). Activation of the TCF/LEF TRE, typically seen in Wnt signalling, will therefore result in EGFP expression (Figure S1A, in Supplementary Material available online at http://dx.doi.org/10.1155/2016/7132529). After transduction, 7 individual clonal lines were isolated by serial dilution and expanded; these clones were tested for their ability to respond to Wnt3a stimulation by flow cytometry (Figure S1B) and analysed by fluorescent microscopy (Figure S1C).  Figure 1(b) demonstrates the responsiveness of the clonal line (A5) selected for further study; minimal expression can be detected when cells are unstimulated, but, upon exposure to Wnt3a, a dose-dependent increase in fluorescence was observed. EGFP fluorescence was then reduced when cells were stimulated with Wnt3a in the presence of IWR-1, a Wnt signalling inhibitor. The ability to respond to Wnt3a was also detectable by flow cytometry, which is more sensitive to low levels of fluorescence. Here, fluorescence was plotted on two green channels to identify cellular autofluorescence, which will be equal in both channels so resulting in events plotted in a straight line. Where positive EGFP expression is present, fluorescent events deflect away from the autofluorescence midline and are captured in a gate. Analyses in this way typically showed that unstimulated cells had a fluorescent population of less than 5% (Figure 1(c)), after cells have been gated to remove dead cells and debris. Upon stimulation with 600 ng/mL Wnt3a, 80% of Y201-Wnt hTERT MSCs were fluorescent (Figure 1(c)). Therefore clone A5 of the Y201 MSC-EGFP reporter cells was used throughout for analyses of Wnt effects in a human MSC model.

### 3.2. Wnt Signalling Is Not Activated in Y201-Wnt hTERT MSCs during Osteogenesis or Adipogenesis in 2D Culture

**Figure 2:**
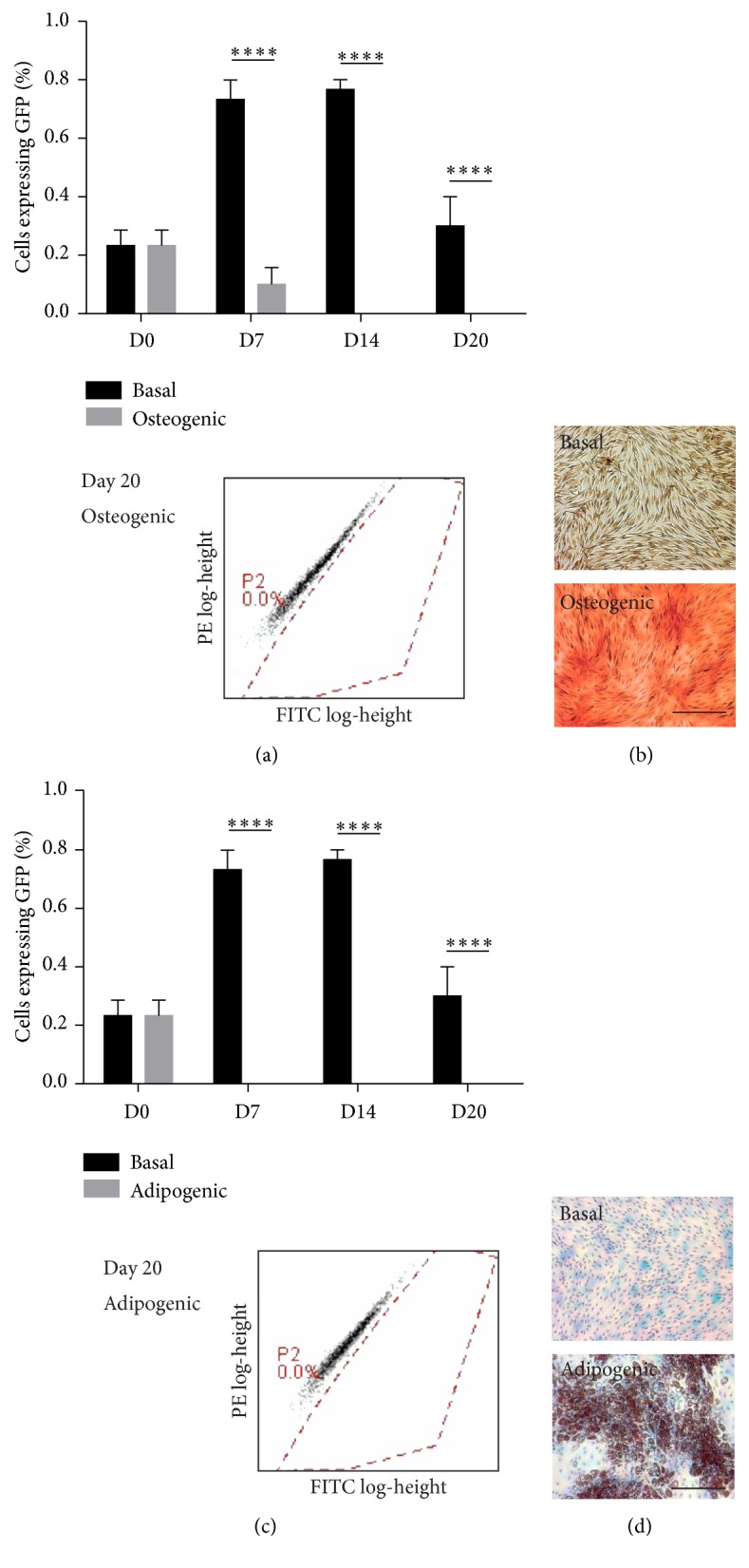
Endogenous Wnt signalling is not activated during 2D MSC differentiation. (a) Percentage of positively expressing human Y201 MSC-EGFP reporter cells during osteogenic differentiation as measured by flow cytometry, ^*∗∗∗∗*^ *p* < 0.0001 versus basal control with representative flow cytometry plot. (b) Alizarin red staining for calcium deposition during osteogenic differentiation. Scale bar = 200 *μ*m. (c) Percentage of positively expressing human Y201 MSC-EGFP cells during adipogenic differentiation as measured by flow cytometry, ^*∗∗∗∗*^ *p* < 0.0001 versus basal control with representative flow cytometry plot. (d) Oil Red O staining for lipid droplet formation during adipogenic differentiation. Scale bar = 200 *μ*m.

Wnt signalling activity is typically implicated in regulating stem cell proliferation and differentiation, particularly in MSCs and skeletal lineages. We therefore sought to determine the status of endogenous Wnt signalling during both osteogenesis and adipogenesis, as an inverse relationship is thought to exist in their lineage specification. Interestingly, we were unable to detect Wnt signalling activation in either differentiation route (Figures 2(a) and 2(c)). Over a 20-day time-course of osteogenic and adipogenic induction, no EGFP-positive cells could be detected by flow cytometry at any stage and they were even significantly below the low EGFP levels in basal undifferentiated conditions at the same time points. Histological staining demonstrated that the Y201-Wnt hTERT MSCs were successfully differentiating and depositing calcium under osteogenic conditions (Figure 2(b)) or producing lipid droplets under adipogenic conditions (Figure 2(d)). It would therefore appear that differentiation of MSCs using typical 2D culture systems does not require activation of endogenous canonical Wnt signalling and the lack of Wnt activation during osteogenesis* in vitro* does not replicate* in vivo* observations.

### 3.3. Endogenous Wnt Signalling Pathway Is Activated in 3D MSC Cultures

**Figure 3:**
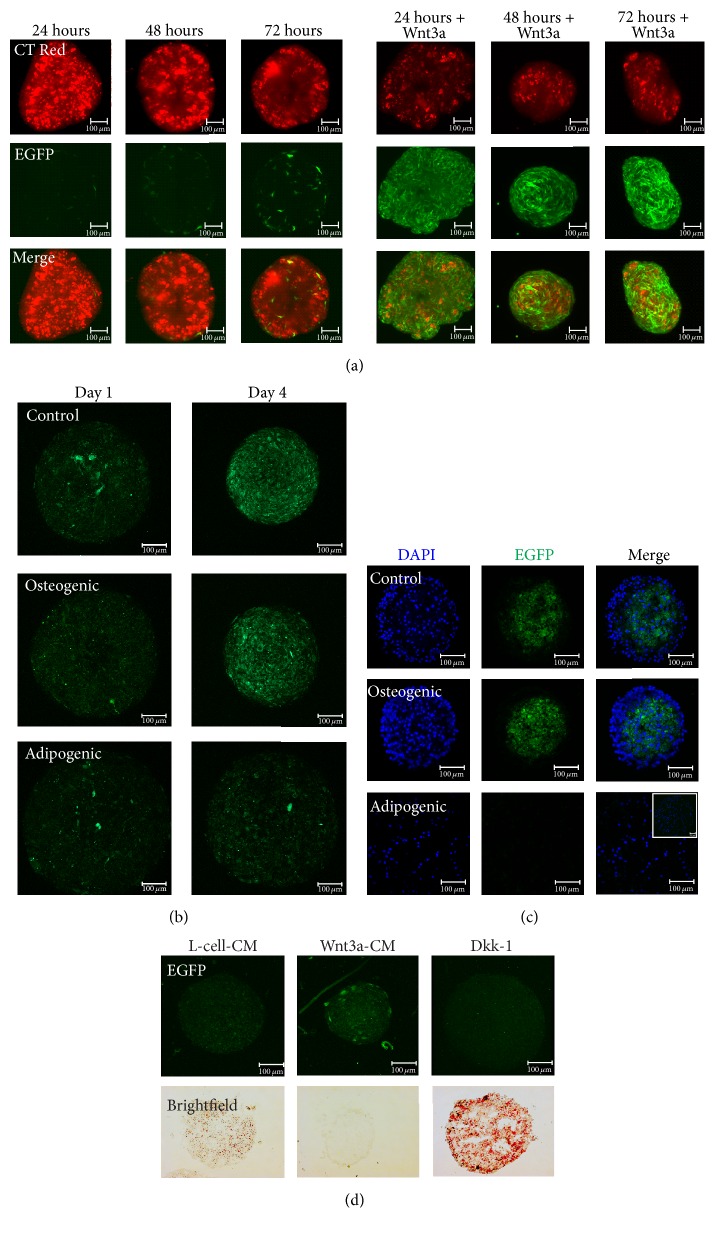
Wnt pathway activation is observed reproducibly in 3D MSC culture and during osteogenic differentiation but not during adipogenic differentiation. (a) Lightsheet microscopy of cell tracker red stained human Y201 Wnt EGFP MSCs showing sporadic activation of Wnt signalling increasing with time in culture, with Wnt3a-treated controls (right panels). Scale bar = 100 *μ*m. (b) Confocal microscopy of murine C3H10T1/2 MSC 3D cultures showing Wnt signalling is active in basal conditions and during osteogenic differentiation but not adipogenic differentiation. Scale bar = 100 *μ*m. (c) Immunofluorescence of sectioned spheroids stained for EGFP demonstrates location of EGFP fluorescence in 3D culture. Scale bar = 100 *μ*m. Adipogenic differentiation causes spheroids to enlarge in size; a lower magnification image of the whole spheroid is included (top right, merge panel). (d) Adipogenic C3H10T1/2 MSC spheroids show inverse relationship between adipogenesis and Wnt signalling by Oil Red O staining.

2D culture systems do not accurately represent the complexity of an* in vivo* system; therefore to determine if a 3D culture system would better replicate* in vivo* Wnt pathway activation we tested the Y201-Wnt hTERT MSCs in a self-aggregating spheroid culture system. Results showed that after 1 day of spheroid formation there was low level, sporadic EGFP expression indicative of endogenous Wnt signalling in individual cells which increased by day 3 (Figure 3(a)). Y201-Wnt hTERT MSCs spheroids were able to respond robustly to Wnt3a stimulation which resulted in the majority of cells in the spheroid expressing high levels of EGFP. Therefore this 3D culture system is suitable for examining Wnt pathway activation.

To enable species-specific comparison with previous* in vivo* studies using mouse models, we used a murine MSC line, CH310T1/2, which also has trilineage differentiation potential. These cells behave similarly to the human Y201-Wnt hTERT MSC cell line in that they are responsive to Wnt in a dose-dependent manner and no Wnt activation could be detected in 2D differentiation studies (results not shown). In basal conditions, sporadic EGFP expression was detected in CH310T1/2 spheroids, which increased over time in culture, similar to the pattern observed in human MSC spheroids, but with a much more widespread distribution, appearing throughout the spheroid (Figure 3(b), control; compare to Figure 3(a)). Active Wnt signalling was also observed in spheroids during early osteogenesis; however TCF-dependent EGFP expression in adipogenic spheroids remained low (Figure 3(b)). To determine more precisely the location of EGFP-positive cells in 3D structures and confirm EGFP expression, spheroids were cryosectioned and immunostained with an anti-EGFP antibody. After 7 days of 3D culture, an EGFP-positive pool was predominantly located in the spheroid centre with EGFP-negative cells at the periphery in undifferentiated and osteogenic sections. Again, no EGFP expression was detected in sections of adipogenic spheroids; it should be noted that adipogenic spheroids increase in size over time due to the accumulation of lipid droplets (Figure 3(c)).

Finally, we determined the effects of exogenous Wnt stimulation on 3D cultures. In undifferentiated control samples endogenous EGFP expression was displayed over the whole spheroid, an effect repressed by the Wnt antagonist, Dkk-1, and retrieved by treating with Wnt3a-CM (Figure S2). However, in agreement with previous results, EGFP expression could not be detected during adipogenic differentiation over a time-course of 7 days (Figure 3(d)). Supplementing the media with Wnt3a-CM induced sporadic EGFP expression in individual cells within the adipogenic spheroid and inhibited adipogenic differentiation as shown by a lack of Oil Red O staining. In contrast, EGFP expression was absent following treatment with the Wnt inhibitor, Dkk-1, which promoted lipid accumulation and increased spheroid size, seemingly accelerating adipogenic differentiation compared to L-cell-CM controls (Figure 3(d)).

## 4. Discussion

Previous studies have identified Wnt signalling as a major developmental signalling pathway, particularly critical in stem cell maintenance and differentiation. However, Wnt function and the onset of its activation are not always clear using typical* in vitro* culture methods, which we have sought to address in MSCs by analysing endogenous and exogenous Wnt signalling in a 3D context.

Human tissues are complex three-dimensional multicellular structures. Recently,* in vitro* techniques have been developed that allow the growth and manipulation of cells in 3D, including the use of cell aggregates such as those examined in this study. The imaging of these aggregates is not always trivial and often dependent on the laser penetration. Previous studies, including ours, have used a multiphoton confocal microscope granting deeper penetration into a sample with less damage as longer wavelengths can be used to excite fluorophores [24–26]. However, imaging in this capacity may still only grant a limited picture of an entire spheroid. New advances in Lightsheet microscopy have employed the use of suspending spheroids in agarose solution allowing the spheroid to be rotated and images to be taken from multiple angles [27, 28]. The combination of reporter cell lines, 3D spheroid culture, and advanced imaging techniques allows for the analysis of signalling at a single cell resolution and offers greater insight into the mechanisms controlling tissue function.

First, we developed stable reporter cell lines in the human Y201 hTERT MSCs and a commonly used murine cell line, C3H10T1/2, both capable of multilineage differentiation* in vitro* when given appropriate stimuli [21, 24, 25]. These cell lines were responsive to exogenous Wnt stimulation with almost undetectable expression of GFP during 2D culture, yet no Wnt signalling activation could be detected during 2D differentiation towards either the osteogenic or the adipogenic lineages. These observations suggest strongly that active Wnt signalling is not a biological requirement using these* in vitro* MSC systems, which contradicts most* in vivo* evidence. It was hypothesised that, by growing MSCs in 3D cultures, the* in vivo* environment is more closely recapitulated in very simplistic terms, which could influence Wnt signalling activity in MSCs compared to conventional 2D methods. Under osteogenic conditions, Wnt signalling was active in a manner similar to undifferentiated spheroids. However, under adipogenic conditions, Wnt signalling was suppressed.

Interestingly, in both undifferentiated and differentiated 3D cultures, Wnt signalling activity appeared to be much more prominent in the centre of the spheroid. Here it would be expected that cells would be subjected to different signalling inputs compared to cells at the surface. Some of these inputs will be altered concentration gradients such as nutrients, signalling molecules, oxygen, mechanical forces, and cytoskeletal tensions [29]. All these factors play a major role in driving cell-contact mediated signalling including the Wnt pathway. This is particularly important considering the locally acting nature of Wnt molecules, which has recently been demonstrated using a 3D* in vitro* culture of intestinal stem cells [30].

To examine these observations further, adipogenic spheroids were treated with medium conditioned from Wnt3a overexpressing murine cells or a Wnt antagonist. Wnt3a activated EGFP expression but only in sporadic, isolated cells under adipogenic conditions; however staining for adipocytes using Oil Red O showed that activating Wnt signalling inhibited adipogenesis in 3D cultures. Moreover, suppressing Wnt signalling using Dkk-1 enhanced adipogenic differentiation in C3H10T1/2 spheroids. Taken together, our results show that Wnt signalling is inactive during and inhibitory to adipogenesis, supporting previous observations. For example, it has been found that Wnt3a decreases the expression of adipogenic transcription factors peroxisome proliferator-activated receptor gamma (PPAR*γ*) and fatty acid binding protein 4 (FABP4) in C3H10T1/2 cells, inhibiting adipogenesis [31]. Our data also suggest that Wnt activation during adipogenesis results in a weak TCF-mediated transcriptional response (observed as low EGFP expression), though this may be sufficiently inhibitory to further adipogenic responses. Alternatively, Wnt3a may be acting through noncanonical mechanisms to regulate adipogenesis as previously described [32].

## 5. Conclusions

We have generated two EGFP reporter MSC lines for the real-time study of Wnt signalling. Using these tools we have demonstrated that Wnt signalling is activated in basal and osteogenic conditions only when examined in 3D culture and not in conventional 2D conditions. In contrast, no Wnt-dependent EGFP expression was detected in adipogenic differentiating spheroids and exogenous stimulation of Wnt signalling had a negative effect on adipogenesis. Our study shows the importance of considering MSC signalling mechanisms in a 3D context to avoid potential discrepancies and apparently contradictory observations.
